# A Hybrid CNN-GRU-SE Forecasting Method for Short-Term Photovoltaic Power Considers AFD and Data Aggregation

**DOI:** 10.3390/e28050511

**Published:** 2026-05-01

**Authors:** Keyan Liu, Dongli Jia, Huiyu Zhan, Jun Zhou, Zezhou Wang, Jianfei Bao

**Affiliations:** 1 China Electric Power Research Institute, Beijing 100192, China; liukeyan@epri.sgcc.com.cn (K.L.); jiadl@epri.sgcc.com.cn (D.J.);; 2 Jiaxing Power Supply Company, State Grid Zhejiang Electric Power Co., Ltd., Jiaxing 314000, China; wangzezhou@jx.zj.sgcc.com.cn (Z.W.);

**Keywords:** adaptive frequency decomposition, beluga whale optimization, CNN-GRU, data aggregation

## Abstract

To enhance the accuracy and robustness of short-term photovoltaic (PV) power forecasting, this paper proposes a novel forecasting method that integrates data aggregation, adaptive frequency decomposition (AFD), modified improved beluga whale optimization (MIBWO), and a CNN-GRU-SE hybrid model. First, the Pearson correlation coefficient and the entropy weight method are combined to screen meteorological features that are strongly correlated with PV power output. Considering the geographical distance, a spatial data aggregation strategy is proposed to exploit the spatial correlation among neighboring PV stations and suppress the output volatility of individual stations. Then, the AFD is adopted to adaptively decompose the PV power series into trend and seasonal components, and the MIBWO algorithm is utilized to optimize the cutoff frequency of AFD and key hyperparameters of the CNN-GRU-SE forecasting model simultaneously. Finally, the SHAP method is employed for model interpretability analysis to quantify the contribution of each feature to the prediction results. Simulation results verified the power forecasting accuracy and robustness of the proposed method. Compared with CNN-GRU and BWO-CNN-GRU-SE, the proposed method reduces MAE by 96.23% and 95.03%, respectively. The method maintains stable performance with sunny and cloudy conditions.

## 1. Introduction

With the accelerating transition of the global energy structure toward low-carbon and clean sources, the development and utilization of renewable energy has become a core strategy to address the energy crisis and environmental challenges [1,2]. PV power generation, featuring wide resource distribution, short construction period and low operation and maintenance costs, has emerged as one of the most important forms of clean energy supply in modern power systems. Amid the continuous growth of PV installed capacity and expanding grid integration, the inherent intermittency, randomness and volatility of PV power generation impose significant impacts on power balance, dispatching operation, security and stability of power grids, considerably increasing the difficulties of grid regulation and accommodation. High-precision PV power prediction technology serves as a critical support to smooth PV power fluctuations, enhance grid operational controllability and new energy accommodation capability, and bears important engineering value for ensuring the stable and efficient operation of power systems with high-penetration PV integration. Current PV power prediction methods are mainly categorized into physical methods and statistical methods [3]. Physical methods rely on the photoelectric conversion mechanism of PV modules and atmospheric physical processes, and establish mathematical models to simulate the mapping relationship between illumination, temperature and other factors and output power for power calculation. However, such methods impose high requirements on model parameters and meteorological data accuracy, involve complicated modeling procedures, and are prone to systematic errors caused by environmental uncertainties. Statistical methods, which do not depend on explicit physical mechanisms, mine the nonlinear correlation between historical meteorological data and power generation data, and implement learning and prediction via data-driven models. They are characterized by simple modeling, strong adaptability and low implementation costs. Mainstream statistical prediction methods mainly include regression analysis, time series analysis, support vector machines and neural networks. With excellent data fitting and generalization performance, these methods have become the mainstream research direction in the field of PV power prediction.

In recent years, the rapid advancement of artificial intelligence has empowered deep learning to surpass the constraints of conventional shallow machine learning models. Deep learning architectures, typified by Convolutional Neural Networks (CNN) [4] and Recurrent Neural Networks (RNN) [5], have gained widespread adoption in short-term photovoltaic (PV) power forecasting. RNNs are inherently suited for time-series processing, they are plagued by long-term dependency problems. To mitigate this issue, Long Short-Term Memory (LSTM) networks incorporate gating mechanisms on top of the RNN structure, enabling selective retention of historical information and effectively alleviating long-term dependency [6]. In Ref. [7], a CNN-LSTM hybrid framework was presented, in which CNN is employed to extract deep nonlinear features and invariant patterns from input data, followed by LSTM for sequential prediction. In Ref. [8], it introduced a combined model using Temporal Convolutional Network (TCN) and LSTM; benefiting from parallel computing capability, TCN avoids typical drawbacks of recurrent structures, resolves gradient-related issues in LSTM, and reduces prolonged training time arising from sequential computation. As a lightweight gated recurrent alternative to LSTM, the Gated Recurrent Unit (GRU) [9] has been extensively utilized in PV power forecasting owing to its fewer trainable parameters and higher computational efficiency. A CNN-GRU-based short-term PV power forecasting model was proposed in [10], which successfully overcomes the limited prediction accuracy of standalone GRU models in PV-related applications. Most existing CNN-GRU forecasting models still have limitations in adaptive feature weighting and critical information extraction. Standard CNN cannot automatically distinguish and enhance important feature channels, leading to insufficient utilization of effective information from PV input data.

To boost the prediction accuracy of hybrid forecasting models, researchers have increasingly integrated modal decomposition techniques and metaheuristic optimization algorithms into model pipelines. In Ref. [11], a unified PV power prediction framework was developed by combining LSTM with Empirical Mode Decomposition (EMD), Kernel Principal Component Analysis (KPCA), and the Sparrow Search Algorithm (SSA). EMD is adopted to decompose environmental parameter time series into feature components with diverse time scales. In Ref. [12], a short-term PV power prediction approach was proposed by fusing optimized Variational Mode Decomposition (VMD) with LSTM, where the optimized VMD is capable of decomposing complex fluctuating components of PV power into relatively independent subseries. A WOA-GRNN-based prediction model was presented in [13], in which the Whale Optimization Algorithm (WOA) is utilized to optimize the key parameters of the Generalized Regression Neural Network (GRNN), effectively improving prediction accuracy and stability.

The inherent intermittency and volatility of PV power generation pose severe challenges to achieving high-precision prediction. Decomposition-based techniques have been widely employed for the preprocessing of PV power time series; conventional decomposition methods suffer from distinct limitations. EMD is plagued by severe mode mixing and significant end effects. While VMD offers a more solid theoretical basis and mitigates the deficiencies of EMD, it still requires manual tuning of the mode number K and penalty factor α, which frequently leads to over-decomposition or under-decomposition. These methods rely on data extrema and fixed parameters, resulting in passive decomposition that fails to adapt to the complex fluctuation characteristics of PV power. Accordingly, an AFD approach is adopted in this work to dynamically separate low-frequency and high-frequency components, which effectively alleviates mode mixing and reduces parameter dependence, making it more suitable for PV power series with diverse fluctuation patterns [14].

Existing PV power forecasting studies mostly focus on single-station prediction, which fails to fully exploit the spatial correlation between adjacent PV stations in the distribution station area. The output of a single PV station is easily affected by local shading, equipment faults and other factors, resulting in strong randomness and volatility that restricts to the complex fluctuation characteristics of PV power. A spatial data aggregation strategy based on geographical distance is introduced in this paper to utilize spatial correlations and stabilize the input data characteristics.

Compared with the hybrid forecasting methods, the individual deep learning models show limited capacity in capturing spatial–temporal features. In this paper, a CNN-GRU-SE hybrid structure is constructed to extract deep spatial features, model long-term temporal dependencies, and emphasize critical features through the SE attention mechanism. However, the hyperparameters of hybrid learning models are usually determined manually, which is inefficient and difficult to obtain the optimal values. Thus, the MIBWO [15] algorithm is adopted to optimize the hyperparameters of the hybrid method, reducing human interference, improving model adaptability, and further enhancing the prediction accuracy.

The main contributions are as follows:

(1) To better select meteorological features, the Pearson correlation coefficient [16,17] and the entropy weight method [18,19] are employed.
(2) Considering the aggregation of power curves from geographically distributed stations, the Haversine formula [20,21] is employed to calculate the geographical distances between different stations. It mitigates the volatility differences among PV power stations, and indirectly reduces prediction errors.
(3) Compared with EMD [11] and VMD [12], the AFD method suppresses the mode mixing effect and reduces parameter dependency, improving the prediction accuracy of PV power.
(4) To further improve the prediction accuracy, the SE attention is used to adaptively enhance the weight of important features and suppress useless information. The MIBWO algorithm is employed to optimize the cutoff frequency of the AFD method and the hyperparameters of the hybrid CNN-GRU-SE method.

The remainder of this paper is organized as follows: Section 2 describes the process of meteorological feature selection using the Pearson correlation coefficient and entropy weight method, as well as data aggregation. The improved whale optimization algorithm is introduced in Section 3. The adaptive frequency decomposition method, the construction of the overall prediction model, and the SHAP analysis are presented in Section 4. Simulation results are provided in Section 5. Conclusions are drawn in Section 6.

## 2. Data Correlation Analysis and Aggregation

### 2.1. Meteorological Feature Selection

The magnitude of PV power output is closely related to meteorological factors. This paper adopts a combined linear and nonlinear approach to comprehensively analyze the impact of meteorological features on PV output.

The Pearson correlation coefficient is employed to calculate the linear correlation between each meteorological factor and PV power output. The Pearson correlation coefficient measures the linear correlation between two variables, *X* and *Y*, and is defined as the ratio of their covariance to the product of their standard deviations [16,17]. This method is applicable to continuous variables, assuming both variables follow a normal distribution. The value of the Pearson correlation coefficient ranges from [−1, 1]. Its calculation formula is presented in (1).

(1) $$\;r=\frac{\sum_{i=1}^{n}\left(X_{i}-\bar{X}\right)\left(Y_{i}-\bar{Y}\right)}{\sqrt{{\sum_{i=1}^{n}\left(X_{i}-\bar{X}\right)}^{2}}\sqrt{{\sum_{i=1}^{n}\left(Y_{i}-\bar{Y}\right)}^{2}}}$$

where *X_i_* and *Y_i_*—the *i*-th variables of *X* and *Y*; $\bar{X}$ and $\bar{Y}$—the means of *X* and *Y*; *n*—sample size; *r*—Pearson correlation coefficient.

To further objectively assess the importance of the selected meteorological features for PV power prediction, this paper adopts the Entropy weight method for feature weighting. Based on the degree of variation in each feature value, this method calculates the objective weights of the features using information entropy [18]. Assuming there are *m* samples and *n* evaluations, the original data matrix is *X* = (*x_ij_*)*_m×n_* [19]. The formulas of the Entropy weight method are as follows:

(2) $$y_{\mathrm{ij}}=\frac{x_{\mathrm{ij}}-\min(x_{j})}{\max(x_{j})\;-\min(x_{j})}$$

(3) $$y_{ij}=\frac{\max(x_{j})-x_{ij}}{\max(x_{j})-\min(x_{j})}$$

(4) $$p_{ij}=\frac{y_{ij}}{\sum_{i=1}^{m}y_{ij}},i=1,\ldots ,m;j=1,\ldots ,n$$

(5) $$S_{j}=-\frac{1}{\ln m}\sum_{i=1}^{m}p_{ij}\ln(p_{ij}),j=1,\ldots ,n$$

(6) $$T_{j}=\frac{1-S_{j}}{\sum_{k=1}^{n}(1-S_{k})},j=1,\ldots ,n$$

where (2) and (3) represent two forms of standardization: (2) corresponds to positive indicators, and (3) corresponds to negative indicators. The standardized data *y_ij_* ∈ [0, 1]; *p_ij_* denotes the proportion of the *i*-th sample under the *j*-th indicator; *S_j_* represents the information entropy of the *j*-th indicator; *T_j_* is the weight of the *j*-th indicator, where 1 − *S_j_* is referred to as the information redundancy or divergence coefficient.

This paper selects actual data from a cluster of PV power stations in a certain region for correlation analysis. The influencing factors include global irradiance, diffuse irradiance, air temperature, air pressure, wind direction and speed, as well as humidity.

**Figure 1:**
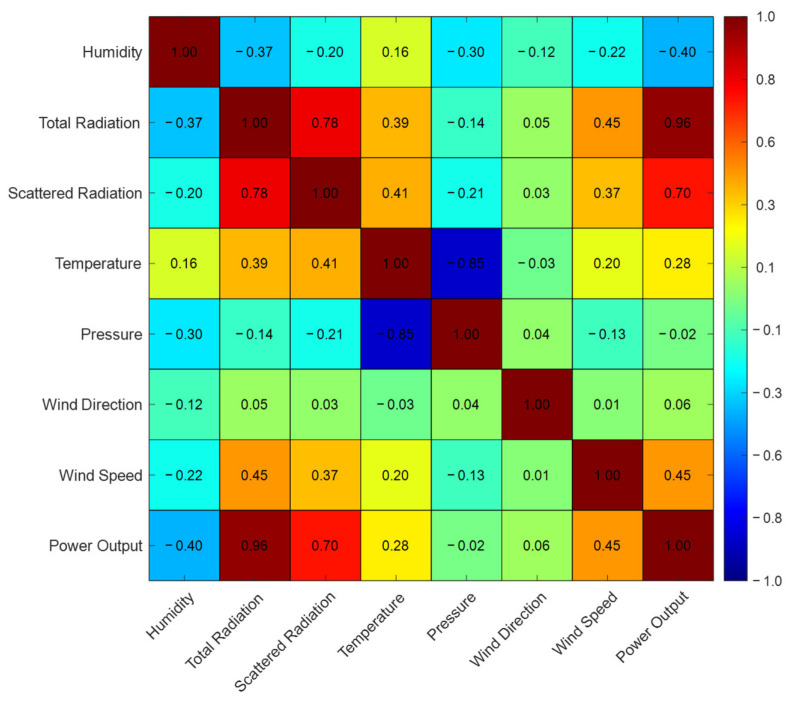
Correlation analysis results.

The correlation coefficients between each meteorological parameter and PV power are shown in Figure 1.

From Figure 1, a global irradiance exhibits the closest relationship with PV power generation, with a correlation coefficient reaching 0.96. Diffuse irradiance is also highly correlated with and has a significant influence on power generation. Air temperature, humidity, and wind speed show a certain degree of correlation with power generation, while atmospheric pressure and wind direction demonstrate a weaker correlation.

**Figure 2:**
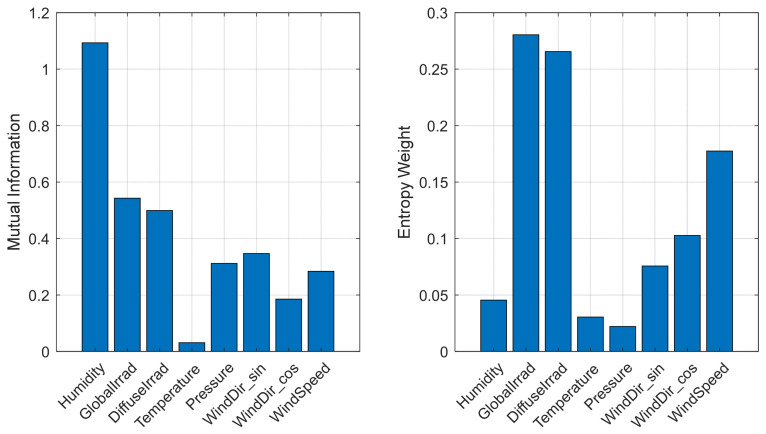
Entropy weights of meteorological features.

From the left panel of Figure 2, it can be observed that humidity exhibits the highest proportion of mutual information. This is attributed to the inclusion of nighttime periods in the dataset, during which both irradiance and PV power output are zero, diluting the relationship between irradiance and PV power. Overall, the weights assigned by the Entropy weight method to features such as temperature, wind direction, and atmospheric pressure, in relation to PV power, are low, indicating that their nonlinear relationships are weak. Although the Entropy weight of temperature is not high, it is retained considering its certain linear correlation.

Therefore, this paper selects global irradiance, diffuse irradiance, temperature, humidity, and wind speed as the meteorological input features for the prediction model.

### 2.2. Transformer Area-Level Data Aggregation and Intra-Transformer Area Data Aggregation

Since PV power generation is influenced by factors such as terrain, component installation tilt angle, and cloud movement, the output of neighboring power stations exhibits spatially similar variation trends. It is considered feasible to aggregate and analyze data from nearby PV power stations within a certain range.

Data aggregation involves calculating the weighted average of meteorological features and PV power generation from different power stations within a specified range, followed by summation to produce a new dataset. This process is fundamentally a form of dataset preprocessing.

The specific method for data aggregation employs the Haversine formula to calculate the distances between power stations within the region [20]. A distance decay function is then applied to convert geographical distances into correlation coefficients [21]. Finally, a weighted average summation is performed based on the correlation coefficients between the power stations. The detailed calculation formulas are as follows:

(7) $$\Delta \phi \;=\;\phi_{i}-\phi_{j}$$

(8) $$\Delta \lambda =\lambda_{i}\;-\;\lambda_{j}$$

(9) $$a=\sin^{2}(\frac{\Delta \phi }{2})+\cos\phi_{1}\cdot \cos\phi_{2}\cdot \sin^{2}(\frac{\Delta \lambda }{2})$$

(10) $$\;c=2\cdot \arctan2\left(\sqrt{a},\sqrt{1\;-\;a}\right)$$

(11) d=R·c

(12) $$\rho_{\mathrm{ij}}=\exp\left(-\frac{d}{\lambda }\right)$$

where $\phi_{i}$ and $\lambda_{i}$ represent the latitude and longitude (in radians) of power station *i*, while $\phi_{j}$ and $\lambda_{j}$ represent the latitude and longitude (in radians) of power station *j*; *R* is the average radius of the Earth (typically taken as 6371 km); *d* is the geographical distance between the two power stations (in km); and $\rho_{\mathrm{ij}}$ is the distance-based correlation coefficient between power station *i* and power station *j*.

After calculating the distance-based correlation coefficients between the power stations, a representative power station is selected that is located near the geographical center of the region. The self-correlation coefficient of the representative power station is $\rho_{\mathrm{ii}}$ = 1, while the correlation coefficients $\rho_{\mathrm{ij}}$ between other power stations and the representative power station are calculated using (12). The formula for calculating the aggregated feature weights of power stations within the region is as follows:

(13) $$w_{j}=\frac{\rho_{ij}}{\sum_{j=1}^{n}\rho_{ij}}$$

Due to the limited geographical scope of a transformer area, it can be assumed that the same meteorological characteristics apply throughout the area. Unlike transformer area-level data aggregation, intra-transformer area data aggregation involves averaging the instantaneous active power and positive active power of multiple loads within the same transformer area at the same moment. This average value is then used to represent the instantaneous and positive active power of the entire transformer area.

## 3. Multi-Objective Improved Beluga Whale Optimization Algorithm

The BWO algorithm is inspired by the collective behavior of beluga whales and their mechanisms of information sharing among individuals. It is characterized by its simple structure, ease of implementation, and high stability. There remains room for improvement in both its convergence speed and solution accuracy.

### 3.1. Population Initialization Based on Chaotic Mapping with Opposite Solutions

A certain search capability is exhibited by the BWO algorithm during its initial phase. The initial individuals, being randomly generated, tend to aggregate together. The algorithm is rendered susceptible to becoming trapped in local optima, from which it cannot escape. To seek the global optimum and expand the search space, population diversity can be enhanced by introducing number sequences that possess irregular properties at the stage of population initialization. Further randomness of individuals in the early iterations is required. The PWLCM chaotic mapping is employed for the improvement of both the randomness and ergodicity of the algorithm’s individuals. The capacity to escape local optima is promoted. The ergodicity of individuals is enhanced during the algorithm’s initial phase. The PWLCM chaotic mapping is presented as

(14) $$m\left(t+1\right)=\left\{\begin{array}{c} \frac{m\left(t\right)}{n},\;0\;\leq \;m\left(t\right)\;<\;n\\ \frac{m\left(t\right)-n}{0.5\;-\;n},\;n\;\leq \;m\left(t\right)\;<\;0.5\\ \frac{1-n-m\left(t\right)}{0.5-n},\;0.5\;\leq \;m\left(t\right)\;<\;1-n\\ \frac{1-m\left(t\right)}{n},\;1-n\;\leq \;m\left(t\right)\;<\;1 \end{array}\right.$$

where *m*(*t*) represents the state value of the chaotic sequence at the *t*-th iteration, and *m*(*t* + 1) denotes the updated state value at the (*t* + 1)-th iteration. The parameter *n* is a critical segmentation coefficient that controls the piecewise structure of the map. In this paper, *n* is set to 0.4, which satisfies the constraint 0 < *n* < 0.5 and ensures the complete chaos of the mapping. The initial state *m*(0) is randomly generated within the interval (0, 1), excluding fixed points such as *n*, 0.5, and 1 − *n* to guarantee the ergodicity of the chaotic sequence. By utilizing PWLCM for population initialization, the search space of the algorithm is expanded, and the randomness and ergodicity of the initial beluga individuals are significantly improved.

Quasi-oppositional learning enables probabilistic updates of individual positions during the algorithm’s iterative process. By leveraging the rich information provided by opposite individuals, it not only further enhances population randomness but also effectively improves the algorithm’s convergence performance. To maintain global search capability while enhancing algorithmic performance, quasi-oppositional learning is integrated to optimize the strategy. The update of beluga individual positions using the quasi-oppositional learning strategy is shown as

(15) $$X_{i,j}^{t+1}=C_{i,j}+\left(X_{i,j}^{o}-C_{i,j}\right)\;\times \;\mathrm{rand}$$

(16) $$X_{i,j}^{t+1}=C_{i,j}+\left(C_{i,j}-X_{i,j}^{o}\right)\;\times \;\mathrm{rand}$$

where $X_{i,j}^{o}$ is the opposite solution of $X_{i,j}$, $C_{i,j}$ is the center value of the upper and lower bounds, and $X_{i,j}^{t+1}$ is the new opposite solution generated by the quasi-oppositional learning strategy.

### 3.2. Dynamic Constrained Local Perturbation Search Mechanism

The transition of the BWO algorithm from exploration to exploitation is determined by a balance factor. Multiple local optima often exist in complex optimization problems. The algorithm’s ability to escape local optima may be hindered by a linearly decreasing strategy. An enhanced global search capability is required during the early stages of the algorithm. A nonlinear convergence factor is introduced for the improvement of the balance factor. This nonlinear convergence factor is shown as

(17) $$B_{f}=B_{0}\left(1-\frac{T}{2T_{\max}}\right)\cdot p$$

(18) $$p=2-2{\left(\frac{\frac{t}{e^{T\max}}-1}{e-1}\right)}^{k}$$

where *k* represents a positive constant, which is adopted to regulate the pace of growth or reduction in the nonlinear convergence factor. During the initial iteration stages of the algorithm, the algorithm is endowed with robust global exploration capability by a greater convergence factor, with a wide search range guaranteed. Iteration counts climb, and the solution draws near to the optimal value. The balance factor is reduced rapidly, with the local optimization capability enhanced.

Considering the complexity of the photovoltaic power prediction optimization problem, multiple comparative experiments verify that a nonlinear convergence factor with *k* = 0.6 achieves an ideal dynamic balance between broad global search in the early stage and rapid local optimization in the later stage. This setting enables the algorithm to sufficiently explore the solution space at the early iterations and prevent premature convergence. It also allows fast convergence to the optimal solution in the late iterations, striking a balance between search efficiency and optimization accuracy. For the univariate optimization task of the cut-off frequency *f_c_* in AFD, and the multivariate joint optimization task of hyperparameters (learning rate, GRUs, number of convolution kernels, etc.) of the CNN-GRU-SE method, the iteration number *T_max_* is 50.

### 3.3. Differentiated Population Optimization Strategy

In the subsequent iterations of the BWO algorithm, beluga individuals deliver the optimal fitness value and move forward to the subsequent iteration cycle during the whale fall stage. Population diversity diminishes as all individuals converge towards the optimal solution. Uneven distribution of the beluga population is observed in the post-initialization phase following each iteration, which elevates the likelihood of the algorithm being trapped in local optima and weakens the effectiveness of convergence precision.

In the search and predation phase of the whale optimization algorithm, the current position update is based on changes in coefficient *A*. If coefficient *A* exceeds the specified range, the current position of the whale individual is randomly updated via distance *D*. This expression is shown as

(19) $$X(t+1)\;=X_{\mathrm{best}}-A\cdot D$$

Inspired by the whale optimization algorithm, and to prevent the BWO algorithm from falling into premature convergence while enhancing its convergence accuracy on multimodal functions, a differentiated population evolution strategy is proposed. It is assumed that weaker individuals in the beluga population perish during activities such as swimming and foraging. At this point, the fittest individual in the population inspects the location of the deceased individual. Since the death position of the beluga is random, the inspection step size of the optimal individual is set as *C*_3_, expressed as

(20) $$C_{3}=C_{\max}-\frac{\Delta \mathrm{Ct}}{T_{\max}}$$

The predation strategy of the whale optimization algorithm can enhance both the optimization capability and convergence speed of the algorithm, the position update of belugas in the whale fall phase of the BWO algorithm is introduced as

(21) $$X_{i}^{t+1}=r_{8}X_{i}^{t}+C_{3}X_{\mathrm{best}}$$

where *r*_8_ is a random number between (0, 1). The probability of whale fall *W_f_* is determined based on the balance factor *B_f_*. If a whale fall occurs, the current optimal individual’s position is updated through the differentiated population evolution strategy. This essentially introduces perturbations to the current optimal value, preventing the population from becoming trapped in local optima.

### 3.4. Optimization Problem Definition

In this paper, the MIBWO algorithm undertakes two core optimization tasks: the optimization of the AFD cutoff frequency and the hyperparameter optimization of the CNN-GRU-SE model. The specific definitions are as follows:

(1) Optimization of AFD Cutoff Frequency

The AFD realizes the adaptive decomposition of photovoltaic power sequences via the cutoff frequency *f_c_*, and the selection of the cutoff frequency directly affects the decomposition performance and subsequent prediction accuracy. The cutoff frequency *f_c_* is taken as the decision variable, and the objective function is constructed to minimize the prediction error of the decomposed sequences. The optimization problem can be formulated as

(22) $$\underset{f_{c}\in [f_{\min},f_{\max}]}{\min}J(f_{c})=\sqrt{\frac{1}{N}\sum_{t=1}^{N}(y_{pred,t}(f_{c})-y_{true,t}{)}^{2}}$$

where *y_pred,t_* *f_c_* denotes the predicted value corresponding to cutoff frequency *f_c_*, *y_true,t_* denotes the true value, *N* is the number of samples, and [*f*_min_, *f*_max_] represents the search range of the cutoff frequency.

(2) Hyperparameter Optimization of the CNN-GRU-SE Model

Model hyperparameters directly affect feature extraction capability and prediction accuracy. The number of CNN kernels *n_cnn_*, the number of hidden layer neurons in GRU *n_gru_*, learning rate *η*, and batch size *b* are taken as decision variables *s* = [*n_cnn_*, *n_gru_*, *η*, *b*]. With the minimization of model prediction error as the objective function, the optimization problem can be formulated as

(23) $$\underset{s\in \Theta }{\min}J(s)=\sqrt{\frac{1}{N}\sum_{t=1}^{N}(y_{pred,t}(s)-y_{true,t}{)}^{2}}$$

where *Θ* denotes the search space of hyperparameters. In this paper, the MIBWO algorithm is adopted to collaboratively optimize the above two optimization tasks, so as to achieve the global optimization of the cutoff frequency and model hyperparameters, and further improve the prediction accuracy.

## 4. Model Construction and Related Principles

### 4.1. Adaptive Frequency Decomposition

To tackle the intrinsic complexity of temporal signal datasets, decomposition techniques are widely employed to partition raw data into more structured sub-components, with predictive accuracy elevated. A self-adaptive frequency decomposition approach, referred to as AFD, is utilized for the analysis of temporal signal sequences, which are transformed into the frequency domain through FFT. A dynamic spectral filter is constructed to automatically partition low-frequency trend constituents and high-frequency seasonal constituents based on the spectral properties of the dataset, with inverse fast Fourier transform (IFFT) implemented on the partitioned constituents to reconstruct the trend sequence and seasonal sequence within the temporal domain. Frequency intervals are distinguished in a self-adaptive fashion by the introduced approach based on the distinct spectral signatures of the dataset. The entanglement of high and low frequencies is effectively prevented, with predictive precision and generalization performance enhanced across diverse data collections. This frequency-domain transform is extensively applied in temporal signal processing workflows.

The FFT is implemented on the input signal sequence, with the sequence transformed from the temporal domain into the frequency domain. Key fluctuations in frequency constituents associated with trend and seasonal characteristics are captured by the model, with a robust foundation established for follow-up decomposition and modeling procedures. The Discrete Fourier Transform (*DFT*) is mathematically formulated as

(24) $$\;X(k)=\mathrm{DFT}[x(t)]=\sum_{n=0}^{L-1}x(t)e^{i\frac{2\pi }{L}\mathrm{nk}},\;k=0,\;1,\;\ldots ,\;L\;-\;1$$

where *L* is defined as an integer power of 2 that specifies the sequence length, *x*(*t*) is taken as the sampled value at time step t within the input sequence *X*, and *X*(*k*) is designated as the Fourier transform outcome.

Dynamic spectral filtering techniques are employed to differentiate high-frequency spectral constituents from low-frequency spectral constituents. Fixed frequency intervals are typically utilized by conventional decomposition techniques for partitioning, a practice that restricts their adaptability to the unique properties of diverse temporal sequences. An adaptive frequency-domain filtering approach is introduced, which dynamically partitions low-frequency and high-frequency constituents based on the spectral properties of the input signal sequence. The decomposition procedure is optimized through alignment with the measured spectral properties, with accurate differentiation achieved between trend (low-frequency) constituents and seasonal (high-frequency) constituents.

For input temporal sequences, FFT is implemented to transform the signal into the frequency domain, with the corresponding frequency-domain representation acquired. The squared amplitude of the complex spectral signal (i.e., the power spectrum) is computed, with the mean value derived across all samples and channels to yield the global frequency power profile, which is formulated as

(25) $$\;P(f)=\frac{1}{B\cdot D}\sum_{b=1}^{B}\sum_{d=1}^{D}|X_{f}^{(b,d)}(f){|}^{2}$$

where *X_f_*^(*b*,*d*)^(*f*) is designated as the complex spectral component associated with the *d*-th feature of the *b*-th sample at frequency *f*.

The cumulative distribution function (CDF) of the power spectrum is utilized to derive a cutoff frequency *f_c_*, with spectral constituents below this threshold primarily associated with the trend component of the input signal and spectral constituents above this threshold primarily linked to seasonal or short-term variations. The power spectrum *P*(*f*) of the input dataset is precomputed to characterize the energy associated with each frequency constituent, with frequency values ordered sequentially from *f* = 0 (DC component) to the maximum frequency *f*_max_. The cumulative energy *E*(*f_i_*) is computed and normalized to a percentage metric *R*(*f_i_*), shown as

(26) $$\;E(f_{i})=\sum_{k=0}^{i}P(f_{k})$$

(27) $$\;R(f_{i})=\frac{E(f_{i})}{\sum_{k=0}^{N}P(f_{k})}\;\times \;100\%$$

In (26), the share of the overall signal energy held within the frequency constituents spanning from the minimum frequency to *f_i_* is represented by *R*(*f_i_*). An energy ratio threshold *θ* is established, which specifies the share of the overall signal energy to be covered by the low-frequency constituents, with the cutoff frequency adaptively derived for distinct data collections. The power spectrum is calculated for each data collection, with the frequency at which the cumulative energy share initially satisfies or surpasses *θ* identified.

(28) $$f_{c}=\min\left\{f_{i}\left|R\left(f_{i}\right)\;\geq \;\theta \right.\right\}$$

Since selecting the energy ratio threshold θ empirically is rather complex, this paper adopts the MIBWO algorithm to determine the energy ratio threshold θ for optimizing the cutoff frequency *f_c_*. After extensive experimental optimization, it is observed that a *θ* value of approximately 0.9 ensures effective separation of high-frequency and low-frequency components in the AFD.

Then, a Gaussian high-pass filter is constructed, with its frequency weighting function formulated as

(29) $$\omega (f)=\exp(-\frac{1}{2}(\frac{f\;-\;f_{c}}{\sigma }{)}^{2})$$

where *σ* is defined as an adjustable smoothing parameter governing the sharpness of the filtering operation. The low-frequency constituent and high-frequency constituent are obtained following the filtering process. These constituents are transformed back to the temporal domain through IFFT, with the trend component and seasonal component yielded. In this paper, σ is set to 0.05, which is determined through extensive comparative experiments to balance the noise suppression and detail retention of the PV power series.

### 4.2. Convolutional Neural Network

CNN is a deep learning model designed to process data with grid-like structures. A typical CNN consists of convolutional layers, pooling layers, and fully connected layers, each with distinct functions. Convolutional layers use multiple kernels that slide over the input sequence, performing convolution operations on local regions to extract features; the parameters of the same kernel are shared across the entire sequence, thereby effectively capturing repetitive patterns. Pooling layers are usually placed after convolutional layers, compressing feature lengths through downsampling (such as max pooling or average pooling) to reduce computational complexity and extract features with scale invariance. In this paper, CNN is employed to perform local feature extraction and deep information mining on the fused multi-dimensional input features. The formulas of the convolutional layer and fully connected layer are presented as

(30) $$y_{t,j}=\sum_{i=1}^{k}x_{t+i-1}\cdot w_{i,j}+b_{j}$$

(31) y=W⋅x+b

where *y_t_*_,*j*_ denotes the *j*-th feature value of the output feature map at time step *t*; *x_t_*_+*i*−1_ represents the element in the input data corresponding to the region covered by the convolution kernel; *k* is the convolution kernel size; *w_i_*_,*j*_ is the convolution kernel weight; *b_j_* is the bias term; *W* is the weight matrix; *x* is the input vector; *b* is the bias term, and *y* is the output.

### 4.3. Gated Recurrent Unit

**Figure 3:**
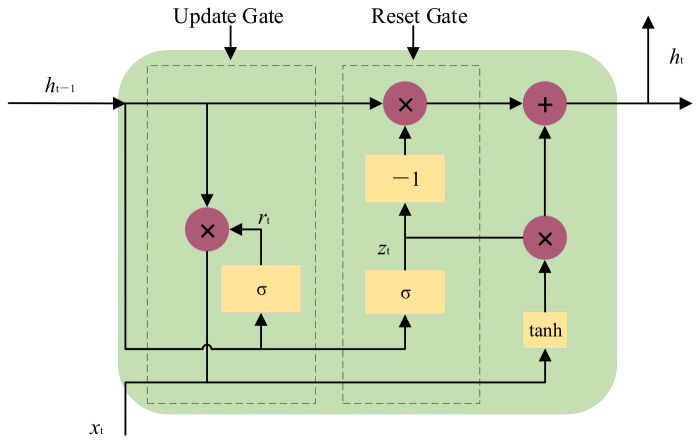
GRU network structure.

The GRU is classified as a specialized variant of the RNN, which is constructed with gated control units. A streamlined structural configuration is adopted by this network, which incorporates solely two gating mechanisms—the reset gate and the update gate. The cell state and hidden state inherent to the LSTM architecture are consolidated into a unified hidden state within the GRU framework. A reduction in computational complexity is realized through this structural design, with the capability to capture temporal dependencies preserved. The corresponding mathematical formulations are presented as follows, with the network architecture depicted in Figure 3.

(32) $$\left\{\begin{array}{ll} & z_{t}=\sigma (W_{z}x_{t}+U_{z}h_{t-1})\\ & r_{t}=\sigma (W_{t}x_{t}+U_{t}h_{t-1})\\ & {\overset{\sim }{h}}_{t}=\tan h(W_{h}x_{t}+U_{h}(r_{t}\;⨂\;h_{t-1}))\\ & h_{t}=(l\;-\;z_{t})h_{t-1}+z_{t}{\overset{\sim }{h}}_{t} \end{array}\right.$$

where *x_t_* is identified as the input dataset; *W_z_*, *W_r_*, *W_h_*, *U_z_*, *U_r_*, *U_h_* are categorized as weight matrices; *h_t_* and *h_t_*_−1_ are designated as the state variables corresponding to the hidden layer at time steps *t* and *t* − 1, respectively; the hyperbolic tangent function is represented by tanh; ${\overset{\sim }{h}}_{t}$ is defined as the intermediate memory state; the Sigmoid activation function is denoted by *σ*; the element-wise multiplication operation is symbolized by ⨂; *z_t_* and *r_t_* are assigned as the state outputs of the update gate and reset gate at time *t*, respectively.

### 4.4. Squeeze-And-Excitation

The Squeeze-and-Excitation (SE) module is an attention mechanism-based module designed to enhance the ability of CNN to capture data features [22,23]. In this paper, the SE module is adopted to weight and strengthen the deep features extracted by the convolutional layers. The workflow of the SE module is as follows:

The squeeze operation begins with global average pooling, which aggregates each channel’s 2D feature map into a compact, channel-wise descriptive vector.

(33) $$z_{c}=\frac{1}{H\times W}\sum_{i=1}^{H}\sum_{j=1}^{W}X_{c}\left(i,j\right),\;c=1,2,\ldots ,C$$

where *z_c_* is the global descriptor scalar for the *c*-th channel; *i* and *j* index the spatial row and column dimensions, respectively; *H* and *W* denote the feature map size; and *C* is the total number of channels. The excitation stage is subsequently performed, in which channel weights are learned using two fully connected layers and non-linear activation functions.

(34) $$s=\sigma \left(W_{2}\delta \left(W_{1}z\right)\right)$$

where *z* is the channel description vector derived from (34), *s* denotes the corresponding channel weight vector, *r* refers to the channel compression ratio, *δ*(·) represents the ReLU activation function, and *σ*(·) stands for the Sigmoid activation function.

The recalibration operation is implemented to remap the learned channel weights onto the original feature maps.

(35) $${\overset{\sim }{X}}_{c}=s_{c}\cdot X_{c}$$

where ${\overset{\sim }{X}}_{c}$ denotes the recalibrated feature of the *c*-th channel, *X_c_* represents the original feature map, and *s_c_* is the corresponding channel weight.

### 4.5. MIBWO-AFD-CNN-GRU-SE Hybrid Prediction Model

**Figure 4:**
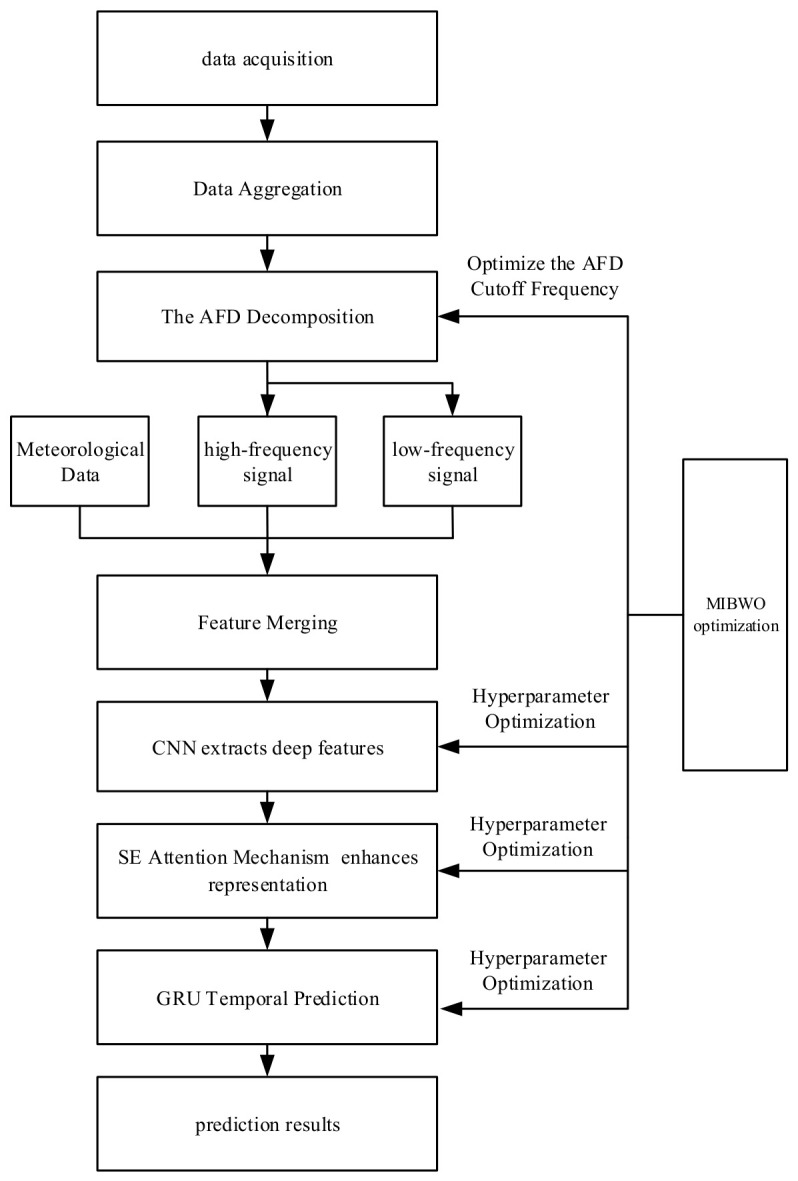
Prediction flowchart of the proposed method.

The prediction flowchart of the proposed method is shown in Figure 4, and steps are as follows:

Step 1: The Pearson correlation coefficient and the Entropy weight method are used to analyze meteorological factors and PV power, selecting those meteorological factors that exhibit high correlation with PV power as features. The distance correlation coefficient between power stations is calculated to perform data aggregation on each feature sequence and PV power sequence. After the dataset preprocessing is completed, the data is fed into the model.

Step 2: Adaptive frequency decomposition is applied to the PV power sequence, and the MIBWO algorithm is used to optimize the cut-off frequencies. The resulting high-frequency and low-frequency components are combined with the original feature set to form a new feature set.

Step 3: The new feature set serves as the input to the combined forecasting model. To simplify power-related features, CNN is employed to perform effective feature extraction on the power-related feature set for dimensionality reduction, thereby obtaining the corresponding key relevant characteristics. The SE attention mechanism is then used to recalibrate the feature values of each channel, enhancing important features while suppressing less significant ones, thus improving the model’s expressive capacity and generalization ability. The MIBWO algorithm is adopted to optimize the model’s hyperparameters, including the learning rate, number of GRU neurons, number of convolution kernels, and the number of neurons in the SE fully connected layer.

Step 4: After the model training is completed, predictions are made on the test set, and the predicted results are subsequently output.

### 4.6. SHAP Feature Analysis

The SHAP (Shapley Additive Explanations) method is one of the most advanced and widely used interpretability tools for machine learning models [24]. Based on the Shapley value principle from cooperative game theory, SHAP explains individual prediction outcomes by allocating contribution values to each feature, while also enabling quantitative analysis of global feature importance, feature marginal effects, and interaction mechanisms [25].

The core idea of SHAP is to treat each feature as a “player” in a cooperative game, where the model’s prediction result is the total payout to be fairly distributed among all features.

Without loss of generality, let the feature set of the sample be *F* = {*x*_1_, *x*_2_, …, *x_n_*}, the prediction function of the model be *f*, then *f*(*X*) is the prediction result of the full feature set *X*, and *f_S_*(*X*) is the prediction result when only the feature subset *S* ⊆ F is retained.

The contribution of feature subset *S* relative to the empty set ∅ is defined as

(36) $$\Delta_{S}(X)=f_{S}(X)\;-\;f_{\emptyset }(X)$$

where *f*_∅_(*X*) is the baseline prediction (usually the average prediction of the model on the training set).

For any feature *x_i_ F*, its SHAP value $\varphi_{x_{i}}$(*X*) (i.e., the marginal contribution to the prediction result) is calculated by the Shapley value formula, which averages the marginal contribution of *x_i_* across all possible feature coalitions.

(37) $$\varphi_{x_{i}}(X)=\sum_{S\subseteq F\backslash \{x_{i}\}}\frac{|S|!\left(|F|\;-\;|S|\;-\;1\right)!}{|F|!}\left(\Delta_{S\cup \{x_{i}\}}(X)\;-\;\Delta_{S}(X)\right)$$

### 4.7. Model Evaluation Metrics

Quantitative analysis of the discrepancy between the forecasted power profile and the measured continuous power profile is conducted. A suite of error assessment metrics is utilized to quantify the extent of discrepancy between the two profiles. Mean Absolute Error (*MAE*), Mean Squared Error (*MSE*), and Root Mean Squared Error (*RMSE*) are incorporated as multi-dimensional indicators for the quantitative evaluation of model performance. The corresponding mathematical formulations are as follows:

(38) $$\;\mathrm{MAE}\;=\;\frac{\sum_{i=1}^{n}\left|X_{i}-Y_{i}\right|}{n}$$

(39) $$\;\mathrm{MSE}=\frac{\sum_{i=1}^{n}(X_{i}-Y_{i}{)}^{2}}{n}$$

(40) $$\;\mathrm{RMSE}=\sqrt{\frac{\sum_{i=1}^{n}(X_{i}-Y_{i})}{n}}$$

where *X_i_* is the predicted value at the *i*-th continuous point, *Y_i_* is the actual value at the *i*-th continuous point, and n is the total duration of PV output in the scenario, i.e., the total number of output data points.

## 5. Simulations

### 5.1. Method Validation for Data Aggregation

To verify the reliability of the data convergence method, transformer area-level forecasting is conducted using data from distributed PV power stations in a certain region. This region includes three PV power stations, and the capacities as well as the longitudes and latitudes of the three stations are shown in Table 1.

From Table 1, the geographical locations of the three photovoltaic power stations are basically adjacent. A data aggregation method is applied to preprocess the datasets of the three stations. The established dataset contains meteorological features and power generation data with a sampling interval of 15 min for power generation records. The division ratio of the training set to the test set is set at 8:2.

**Figure 5:**
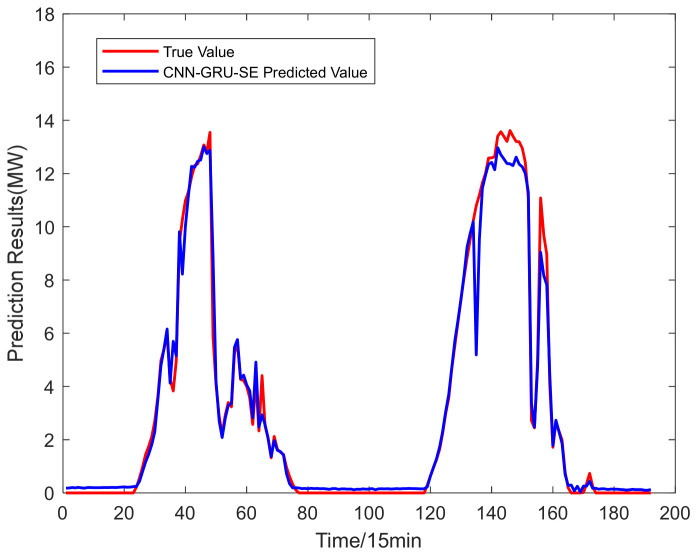
Forecast chart of aggregated PV power stations.

**Figure 6:**
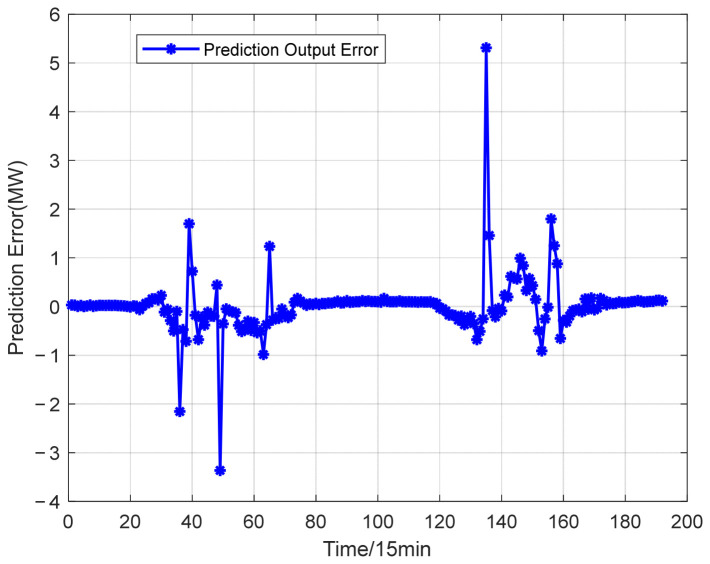
Prediction error of aggregated PV power stations.

The power prediction curves of the test set after data aggregation are presented in Figure 5. The test set is composed of continuous two-day power generation data of a single power station, with a full time coverage from 0:00 to 24:00. The distribution of prediction errors is illustrated in Figure 6. To fully reflect the improvement of prediction accuracy brought by data aggregation, the CNN-GRU-SE model is employed for photovoltaic power forecasting. Prediction errors obtained from single-station data and aggregated multi-station data are summarized in Table 2.

From the results, compared with the forecast outcomes of individual power stations, the predictions obtained under data aggregation are closer to the actual values. The prediction errors of the aggregated power stations, determined through a combination of three correlation calculation methods, are lower than those of the other stations. Compared with single-station prediction, the proposed aggregation method considers the spatial correlation among adjacent stations, effectively suppresses the data fluctuations caused by local shading, equipment anomalies and other factors, and improves the quality and stability of input data.

**Figure 7:**
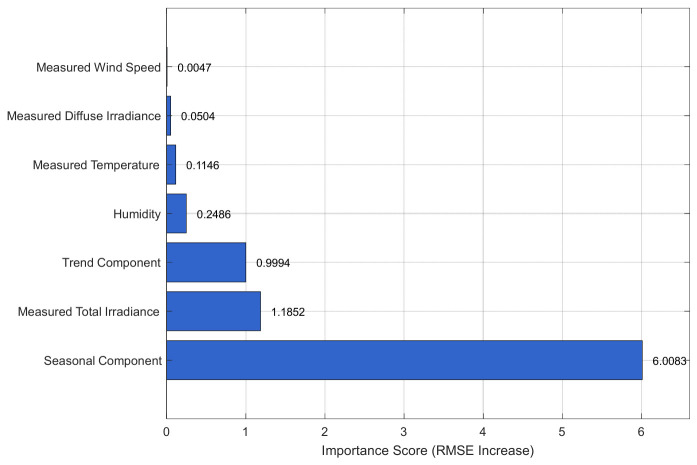
Feature analysis chart.

To further explore the relationship between PV power and meteorological features, as well as the seasonal and trend components obtained from frequency decomposition, this paper employs the SHAP algorithm to conduct an interpretability analysis of PV power generation. Figure 7 presents the meteorological features and frequency decomposition components, with the vertical axis representing feature names and the horizontal axis representing the corresponding Shapley values of the features. From Figure 7, the seasonal component obtained from frequency decomposition has the highest contribution, while the trend component and irradiance among the meteorological features also account for a significant proportion in the prediction.

**Figure 8:**
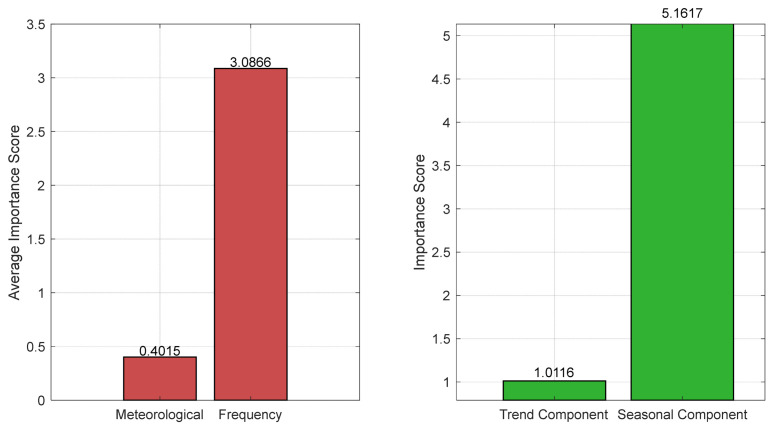
Feature importance results.

As shown in Figure 8, the left panel of Figure 8 presents the average importance scores of meteorological features and frequency decomposition features in the prediction. Among these, the frequency decomposition features significantly outweigh the meteorological features, indicating that the trend and seasonal components extracted from the power series contribute substantially to PV power forecasting.

The right panel of Figure 8 compares the importance of the trend component and the seasonal component obtained through adaptive frequency decomposition. The importance score of the seasonal component is approximately five times that of the trend component. This suggests that the seasonal component, which represents high-frequency periodic fluctuations in the power series, has a substantial impact on PV power prediction. This finding aligns closely with the physical characteristics of PV power generation: PV output is highly influenced by instantaneous changes in irradiance and exhibits strong diurnal cycles and weather-scale fluctuations. Consequently, the seasonal component (high-frequency part) serves as the most critical predictive signal, while the trend component, which primarily reflects medium- to long-term variations, plays a comparatively weaker role in short-term forecasting (e.g., hours to days ahead).

**Figure 9:**
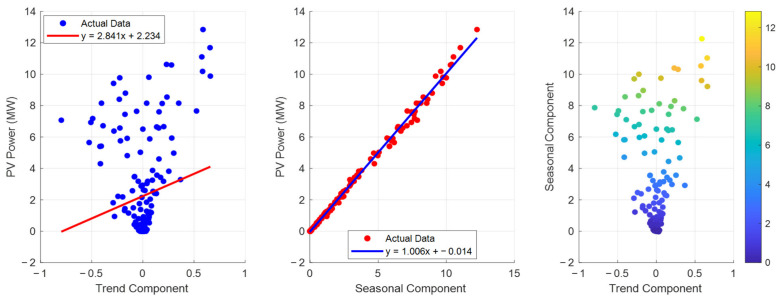
Impact of different components on PV power.

The left panel of Figure 9 illustrates the correlation between the trend component and PV power, while the middle panel shows the correlation between the seasonal component and PV power. It is evident from the figure that the seasonal component exhibits a strong positive correlation with PV power; however, the fitted line has a relatively small slope of 1.006. In contrast, the fitted line for the trend component has a larger slope of 2.841. This indicates that the trend component serves as the primary driving force behind variations in PV power, whereas the seasonal component plays a modulating role.

### 5.2. Evaluation of Algorithm Optimization Effectiveness

**Figure 10:**
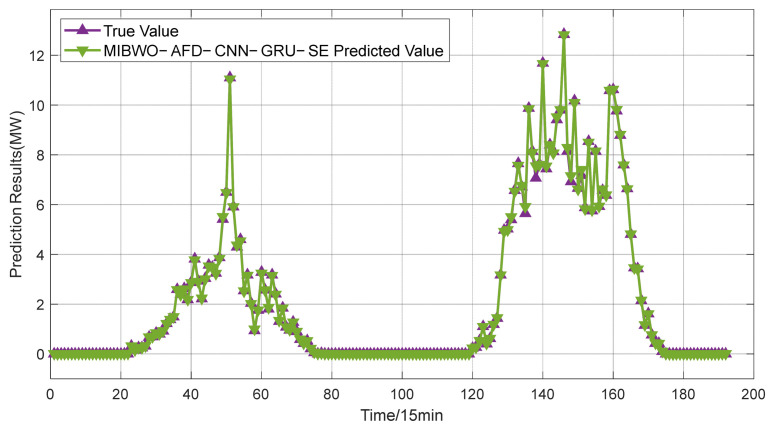
Prediction results of MIBWO-AFD-CNN-GRU-SE.

**Figure 11:**
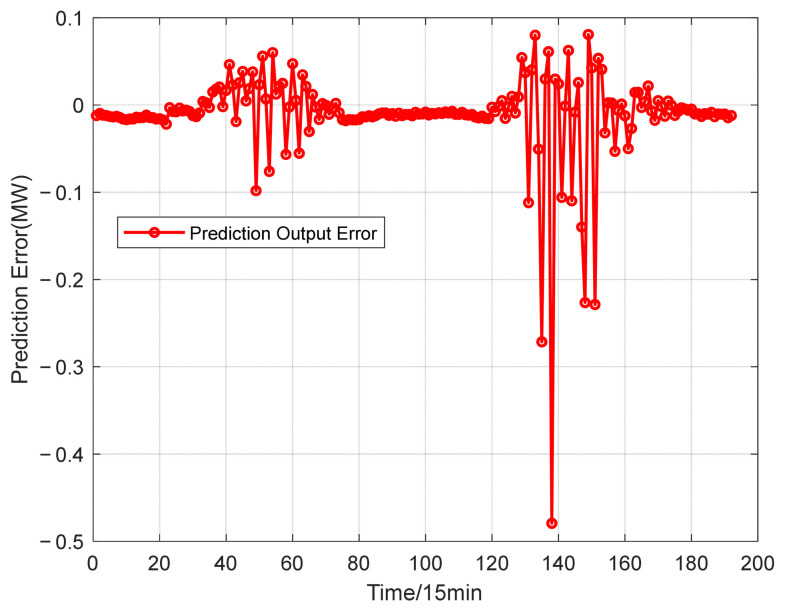
Forecast errors of MIBWO-AFD-CNN-GRU-SE.

To validate the reliability of the proposed MIBWO-AFD-CNN-GRU-SE model in photovoltaic power prediction, prediction results derived from various comparative models are adopted for quantitative comparison. The prediction performance of the MIBWO-AFD-CNN-GRU-SE model is presented in Figure 10. The training set consists of aggregated multi-station data, and the test set selects two-day operational data from a certain power station. The time range covers the whole period from 0:00 to 24:00. The corresponding prediction errors are illustrated in Figure 11. Prediction errors of all comparative models are summarized in Table 3.

From Table 3, the CNN-GRU-SE model achieves an 8.18% reduction in MAE compared with the CNN-LSTM-SE model, while the MSE and RMSE exhibit slight increases. Compared with BWO-CNN-GRU-SE method, the MIBWO-CNN-GRU-SE method achieves a 14.26% reduction in MAE, a 21.39% decrease in MSE, and an 11.34% reduction in RMSE with faster optimization speed observed. When an adaptive frequency decomposition is incorporated into the MIBWO-CNN-GRU-SE method, the prediction accuracy is improved, with MAE reduced by 94.21% and RMSE decreased by 94.80%.

**Figure 12:**
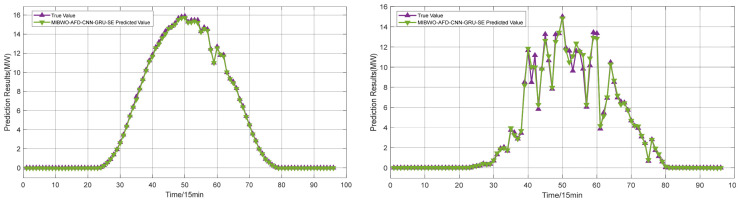
Different weather forecast results.

To further verify the robustness of the proposed MIBWO-AFD-CNN-GRU-SE method under different weather conditions, the prediction performance of the model is tested on three typical weather scenarios: sunny and cloudy days. The 24 h photovoltaic power prediction results are shown in Figure 12. The prediction error is shown in Table 4.

On the left side of Figure 12 are the prediction results for sunny days, with an MAE of 0.0595 MW and an RMSE of 0.098718 MW. The right side shows the prediction results for cloudy days, with an MAE of 0.15996 MW and an RMSE of 0.30367 MW. Power curves under cloudy conditions exhibit more drastic fluctuations in contrast to sunny weather. Higher prediction difficulty is induced accordingly, and much larger prediction errors are obtained in cloudy scenarios. The MAE of cloudy days increases by 104.45% compared with that of sunny days. Overall, MAE values yielded by the proposed model are controlled below 0.2 MW under both sunny and cloudy conditions. Reliable and satisfactory prediction performance is guaranteed in different weather scenarios.

From the previous analysis, the AFD method suppresses the mode mixing effect and reduces parameter dependency. The CNN-GRU-SE hybrid model integrates the spatial feature extraction capability of CNN, the temporal dependence modeling capability of GRU, and the feature enhancement capability of the SE attention mechanism, which can make up for the spatiotemporal features limitations of a single model. The global optimization of model hyperparameters by the MIBWO algorithm avoids the subjectivity of manual parameter tuning and further improves the generalization ability and prediction accuracy of the model.

## 6. Conclusions

To address the intermittency, volatility and low forecasting accuracy of short-term PV power generation, this paper proposes a hybrid short-term PV power forecasting model based on spatial data aggregation and the MIBWO-AFD-CNN-GRU-SE model. A series of data analyses, mechanism verifications and comparative simulations are carried out, and the main conclusions are as follows:

(1) To facilitate the observation of the influence of various meteorological factors on PV power generation, the Pearson correlation coefficient and the Entropy weight method are used to calculate the correlation between each meteorological factor and PV power generation, thereby selecting prediction features with relatively high correlation.
(2) In view of the spatial correlation characteristics of PV power station output across different transformer areas, a data aggregation method based on geographical distance is used. This method effectively exploits the spatial correlation characteristics of PV output, reducing the impact of the randomness and volatility of individual data on prediction. Compared with the prediction using single-station data, the prediction error MAE is reduced by at least 21.42% after data aggregation.
(3) The AFD method is capable of adaptively distinguishing frequency bands based on the specific spectral characteristics of the data, thereby avoiding the confounding of high-frequency and low-frequency components and ultimately improving prediction accuracy.
(4) The MIBWO algorithm has better optimization effects. MIBWO realizes the collaborative optimization of AFD cutoff frequency and CNN-GRU-SE hyperparameters, which overcomes the problems of slow convergence and is easy to fall into local optimum of the standard BWO.
(5) Compared with CNN-GRU and BWO-CNN-GRU-SE methods, the proposed method reduces MAE by 96.23% and 95.03%, respectively, achieving an MAE of 0.02519 MW. Under sunny and cloudy conditions, the method maintains stable performance.

Future research may further expand the coverage of the dataset to include PV power generation data under extreme weather conditions. By exploring multi-model fusion strategies and integrating multi-source heterogeneous data, the adaptability and prediction accuracy of the model in complex scenarios can be significantly improved.

## Figures and Tables

**Table 1:** Power Station Information Data.

Power Station	Capacity (MW)	Longitude	Latitude
PV1	17	114.19887° E	38.05728° N
PV2	20	114.11494° E	38.10956° N
PV3	35	114.1236° E	38.2355° N

**Table 2:** Deviation of prediction under different conditions.

Prediction Method	MAE/MW	RMSE/MW	MSE
Only PV1	0.34999	0.74517	0.55528
Only PV2	0.40986	0.73268	0.53681
Only PV3	0.6457	0.8548	0.73068
Aggregated prediction	0.27502	0.60213	0.36256

**Table 3:** Prediction deviation of different methods.

Prediction Method	MAE/MW	RMSE/MW	MSE
CNN-GRU	0.66741	1.4731	2.1699
CNN-LSTM-SE	0.61785	1.2064	1.4555
CNN-GRU-SE	0.56732	1.2521	1.5678
BWO-CNN-GRU-SE	0.50703	1.1794	1.3911
MIBWO-CNN-GRU-SE	0.43474	1.0457	1.0936
MIBWO-AFD-CNN-GRU-SE	0.02519	0.0544	0.00296

**Table 4:** Prediction error for sunny and cloudy conditions.

Weather Conditions	MAE/MW	RMSE/MW	MSE
Sunny	0.0595	0.098718	0.0097453
Cloudy	0.15996	0.30367	0.092213

## Data Availability

The data are contained within this article.
